# Feasibility of external rhythmic cueing with the Google Glass for improving gait in people with Parkinson’s disease

**DOI:** 10.1007/s00415-016-8115-2

**Published:** 2016-04-25

**Authors:** Yan Zhao, Jorik Nonnekes, Erik J. M. Storcken, Sabine Janssen, Erwin E. H. van Wegen, Bastiaan R. Bloem, Lucille D. A. Dorresteijn, Jeroen P. P. van Vugt, Tjitske Heida, Richard J. A. van Wezel

**Affiliations:** Biomedical Signal and Systems Group, MIRA Institute for Biomedical Technology and Technical Medicine, University of Twente, P.O. 217, 7500 AE Enschede, The Netherlands; Department of Rehabilitation, Radboud University Medical Center, P.O. Box 9101, HB Nijmegen, The Netherlands; Department of Neurology, Radboud University Medical Center, P.O. Box 9101, 6500 HB Nijmegen, The Netherlands; Department of Rehabilitation Medicine, MOVE Research Institute Amsterdam, VU University Medical Center, Boelelaan 1117, 1081 HV Amsterdam, The Netherlands; Department of Neurology, Medisch Spectrum Twente, P.O. Box 50 000, 7500 KA Enschede, The Netherlands; Department of Biophysics, Donders Institute of Brain, Cognition and Behaviour, Radboud University, P.O. Box 9010, 6500 GL Nijmegen, The Netherlands

**Keywords:** External cueing, Gait, Freezing of gait, Smartglasses, Assistive devices

## Abstract

New mobile technologies like smartglasses can deliver external cues that may improve gait in people with Parkinson’s disease in their natural environment. However, the potential of these devices must first be assessed in controlled experiments. Therefore, we evaluated rhythmic visual and auditory cueing in a laboratory setting with a custom-made application for the Google Glass. Twelve participants (mean age = 66.8; mean disease duration = 13.6 years) were tested at end of dose. We compared several key gait parameters (walking speed, cadence, stride length, and stride length variability) and freezing of gait for three types of external cues (metronome, flashing light, and optic flow) and a control condition (no-cue). For all cueing conditions, the subjects completed several walking tasks of varying complexity. Seven inertial sensors attached to the feet, legs and pelvis captured motion data for gait analysis. Two experienced raters scored the presence and severity of freezing of gait using video recordings. User experience was evaluated through a semi-open interview. During cueing, a more stable gait pattern emerged, particularly on complicated walking courses; however, freezing of gait did not significantly decrease. The metronome was more effective than rhythmic visual cues and most preferred by the participants. Participants were overall positive about the usability of the Google Glass and willing to use it at home. Thus, smartglasses like the Google Glass could be used to provide personalized mobile cueing to support gait; however, in its current form, auditory cues seemed more effective than rhythmic visual cues.

## Introduction

People with Parkinson’s disease (PD) commonly experience gait disturbances characterized by decreased stride length and walking speed and increased cadence [17]. Ultimately, freezing of gait (FOG), “an episodic inability to generate effective stepping,” might emerge [7]. FOG typically occurs during gait initiation or turning and is a main risk factor for falling [21]. Together, these motor symptoms severely diminish the quality of life for people with PD [15].

External visual or auditory cues like transverse lines on the floor or a metronome have been shown to alleviate gait impairments and FOG [12, 22]. However, as the carryover effects of home training programs decreased considerably after the training period [13, 19], there is a need for mobile devices that can provide cueing ‘on demand’. Several portable cueing devices have been developed and tested in laboratory settings with promising results, including ‘walking glasses’ with a limited array of programmable light emitting diodes (LEDs) that simulate optic flow [6], rhythmically flashing LEDs [19], or LEDs that project virtual fixed lines [14]. The recent introduction of smartglasses by major technological companies like Google and Microsoft makes mobile personalized cueing accessible for a wider audience. Smartglasses share many features with a smartphone (e.g., GPS, WiFi, accelerometers, and audiovisual output), but its displays can be conveniently worn like conventional glasses, offering greater possibilities for cueing such as three-dimensional cues. Moreover, hands-free interfaces like voice and gesture control increases the usability of these wearable displays for people with PD. As mobile technology advances, smartglasses may become a feasible, cost-effective and socially acceptable way to self-manage gait-related symptoms of PD. However, systematic studies on the efficacy and usability of such devices are lacking.

To investigate the feasibility of smartglasses as mobile cueing devices, we assessed the effects of cueing with the Google ‘Glass’ on gait performance in a randomized laboratory study. Amongst the smartglasses currently on the market, Glass best fitted the user requirements derived from our recent survey on smartglasses applications for PD [32]. We hypothesized that the use of visual and auditory cues delivered by the Glass would lead to reduced FOG episodes, increased stride length and walking speed, and decreased cadence and stride length variability during walking trials.

## Methods

### Patient selection

Participants (*N* = 12) were recruited by neurologists (LD, JV) of the Medisch Spectrum Twente. Potential participants were given written information about the study, adequate time to consider participation, and the opportunity to ask questions. All subjects were diagnosed according to the UK Brain Bank criteria [8] and had a history of FOG (minimum of two events per day), as verified by the New Freezing of Gait Questionnaire (N-FOGQ) [20] with a score of 3 on question 2. Participants must be able to walk 20 m over a flat surface without walking aids and were excluded if they had significant cognitive impairments, based on a Frontal Assessment Battery (FAB) [5], other comorbidities that impaired gait, or visual impairments that prevented use of Glass (prescription glasses were allowed). Prior to testing, the subjects were clinically assessed (JN, ES) with the Movement Disorder Society Unified Parkinson’s Disease Rating Scale (MDS-UPDRS) part III [8], the N-FOGQ, and FAB (see Table 1 for clinical scores). The experiments were scheduled, so that measurements were performed while the participants were in an end-of-dose state, on average 3.04 ± 0.84 h after their last medication intake.

**Table 1:** Clinical characteristics of the subjects (*N* = 12) including scores for the Unified Parkinson’s disease rating scale Part III (UPDRS III, score/132), Hoehn and Yahr (score/5), New Freezing of Gait Questionnaire (N-FOGQ, score/33), Frontal Assessment Battery (FAB, score/18) and daily levodopa dosage

	Mean ± standard deviation	Range
Age	66.8 ± 6.8	53–78
Gender	*F*(*N* = 3), *m*(*N* = 9)	
Disease duration (years)	13.6 ± 6.7	6–24
UPDRS-part III	35.2 ± 10.6	17–54
HY-stage	2 (*N* = 8), 3 (*N* = 4)	2–3
N-FOGQ	22.1 ± 5.1	13–31
FAB	15.7 ± 2.2	11–18
Daily levodopa dosage (mg)	809.1 ± 320.0	200–1200

Higher scores for the UPDRS, HY and N-FOGQ reflect worsening disability while low scores for FAB correspond to poorer performance

Other medications taken on the day of testing (daily dosage in mean ± standard deviation) included symmetrel (233.3 ± 115.5 mg, *N* = 3), rotigotine patch (8.0 ± 2.8 mg, *N* = 2), parlodel (15 ± 0.0 mg, *N* = 2), ropinirole (16 ± 5.7 mg, *N* = 2), elderpryl (10 mg, *N* = 1), comtan (600 mg, *N* = 1), rivastigmine (6 mg, *N* = 1), fluvoxamine (50 mg, *N* = 1), pramipexole teva (1.05 mg, *N* = 1), entacapone (800 mg, *N* = 1), tamsulosin (0.4 mg, *N* = 1), clopidogrel (75 mg, *N* = 1), oxazepam (20 mg, *N* = 1), macrogol (10 mg, *N* = 1), simvastatin (20 mg, *N* = 1), metoprolol (50 mg, *N* = 1), aspirin (80 mg, *N* = 1), allopurinol (100 mg, *N* = 1), omeprazole (20 mg, *N* = 1), and carbasalate calcium (100 mg, *N* = 1)

### Cueing application

**Fig. 1:**
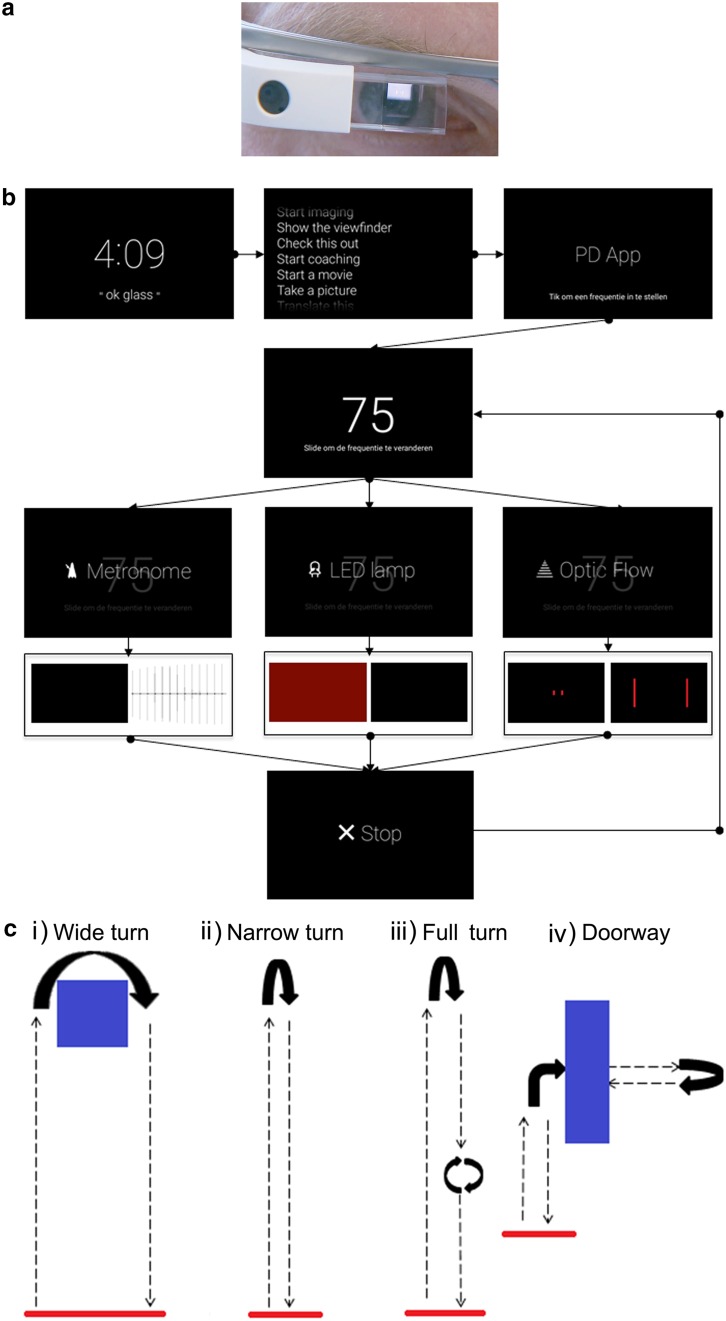
**a** A transparent prism mounted on the *top right* of the frame of the Google Glass displayed visual cues such as optical flow. **b** Flow diagram of the cueing app: The app was voice activated using the prompt “OK glass” followed by choosing “Start coaching” from the list of possible actions. From the main menu of the “PD App,” users could scroll, tap, or swipe to select a desired cueing frequency and choose the type of cue to provide. The app could be stopped at any time. **c** Walking courses: *i*–*iv* 10 m walk forward and back with a *i* wide or *ii*, *iii* narrow U-turn and a *iii* full 360° turn halfway back. *iv* 2 m walk with a right turn through a doorway and turning 180° to walk back

Using Android Studio, we developed an app for Glass (Explorer version 2, XE 22.0, Android 4.0+) (Fig. 1a) that delivered three possible audiovisual cues (metronome, flashing light (LED), or optic flow) according to a desired frequency (range = 50–150 cues/min) (Fig. 1b). The metronome produced a rhythmic auditory beat without any visual display. Selecting the LED function caused the screen to rhythmically flash on and off. The optic flow generated vertically oriented lines on both sides of the screen that moved forward at a fixed speed (in lines/min).

Participants first familiarized themselves with Glass, using voice actions and touch gestures to scroll through menus, choose apps, and input app parameters. Next, the desired walking frequency was determined by counting the number of steps the subject walked in 10 s at a comfortable speed. The subjects fine-tuned the cueing frequency for the app according to their preferred walking speed by performing one to two test runs on a 10-m walk for each of the three different cues. Participants were instructed to synchronize their steps to the rhythm of the cues. For example, the subjects were asked to take one step for each beat they heard of the metronome. For the LED, participants were instructed to take a step whenever the screen flashed off and another when the screen flashed on. In the case of the optic flow, participants were instructed to step with their left foot when a moving bar appeared on the left side of the screen and to step with their right foot when a bar appeared on the right side of the screen. The selected cueing frequency was on average 106.1 ± 11.5 (range = 80–124) steps/min. The same frequency was used for both visual and auditory cues throughout the walking trials.

### Walking trials

Testing was performed at the Experimental Center for Technical Medicine at the University of Twente. Each experiment was conducted over approximately 2.5 h with 1 h allotted for the gait measurements. While wearing Glass, the subjects performed a series of walking tasks on four different walking courses (Fig. 1c) in combination with four cueing conditions (no cue, metronome, LED, and optic flow). During the ‘wide turn’ course, the participants walked 10 m forward, made a wide 180° U-turn around a chair, and walked back to the starting point. On the ‘narrow turn’ course, a narrow 180° U-turn is performed instead. The ‘full turn’ course involved an additional 360° turn halfway on the walking course on the way back. For the doorway course, participants walked 2 m, turned 90°, walked through an open doorway, and turned 180° to head back to the starting point. The 16 different cue-course combinations were tested using a randomized crossover design. All combinations were tested twice per patient. Additional trials, up to four in total for each combination, were performed at the end of the session if time allowed. On average, 5.2 ± 3.7 additional trials were performed per subject. Prior to the measurements, the order of the walking courses and cueing conditions were predetermined using a random number generator without replacement by ES. First, the order of the walking course was determined by consecutively generating four numbers between one and four, corresponding to wide turn, narrow run, full turn, or doorway walking course. Next, the order of the four cueing conditions within each walking course was similarly generated.

### Freezing of gait analysis

The walking trials were video recorded for post hoc analysis of FOG. Cameras were placed at the start and midpoint (i.e. location of the 180° U-turn) of the walking course so that the videos were oriented along the anterior–posterior axis. Two independent experienced raters (JN, ES) blindly scored the videos for the number and duration of FOG during each trial and noted the activity (e.g. turning or walking straight) associated with each FOG episode [7, 20]. Prior to consensus, the raters reached a high degree of agreement for the presence of FOG within each trial (97 % agreement, Cohen’s kappa = 0.83). Disagreements between raters were resolved through discussion.

### Kinematic analysis

**Fig. 2:**
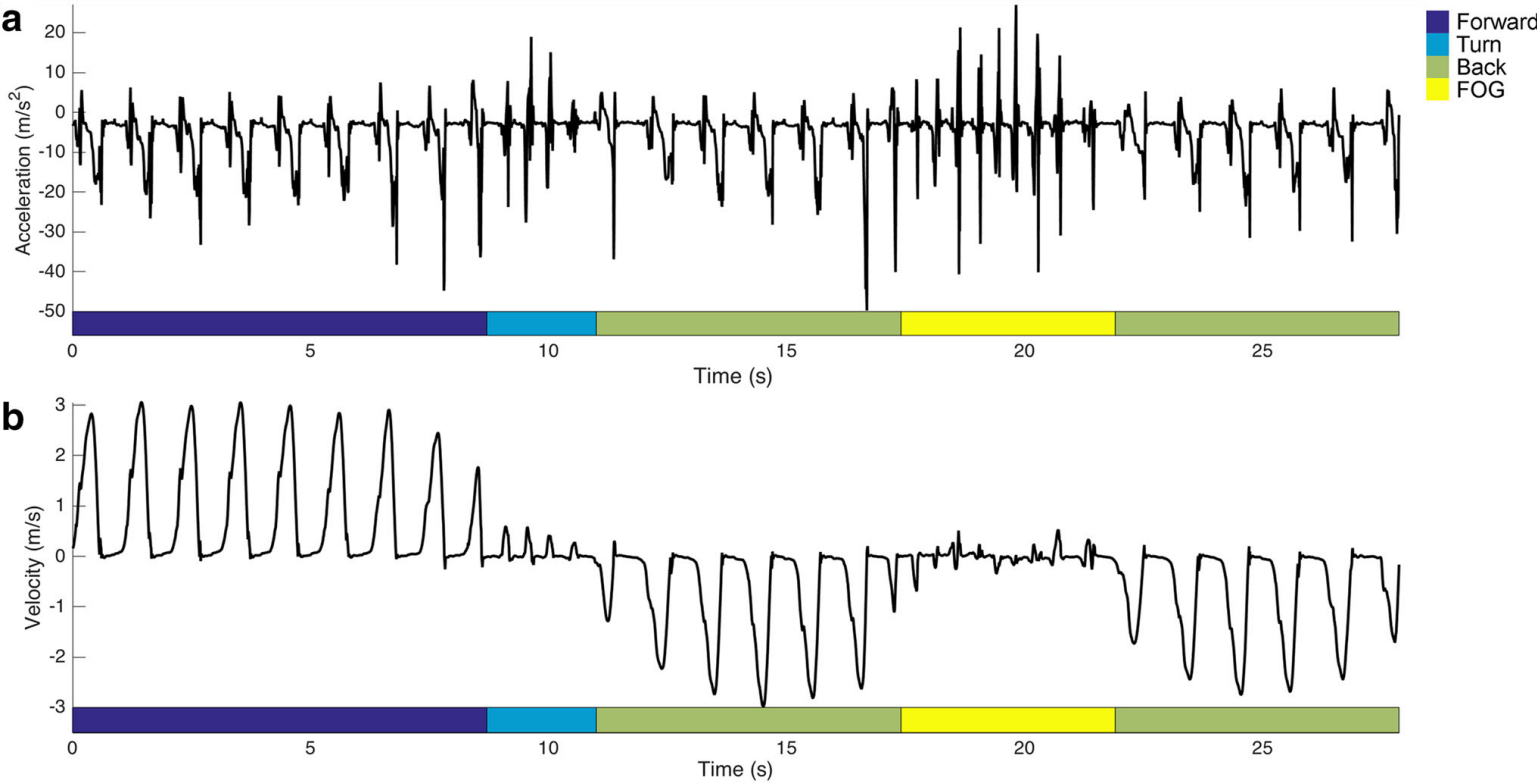
Typical **a** acceleration and **b** velocity waveforms recorded at the feet in the anterior–posterior orientation during a walking trial. The *colored bars* below the waveforms indicate the type of activity performed at each time point, including walking forward towards the midpoint, turning 180°, walking back the to starting positions, and FOG

Motion data were collected using an MVN motion capture suit (Xsens, Enschede, the Netherlands) in the lower body configuration (motion data for one patient was missing due to equipment failure). Seven MTx inertial measurement units containing three-dimensional gyroscopes, accelerometers, and magnetometers were attached to the pelvis (sacrum), the upper legs (lateral side of the femoral shaft above the knee), lower legs (medial surface of the tibia), and both feet (tarsus) with adhesive straps (refer to Fig. 1 of [24] for an illustration of the placement of the sensor units). To maintain comfort during testing, subjects were asked beforehand to wear casual clothing. A calibration procedure in a known *N*-pose (arms neutral besides the body in an upright position) was performed to determine the orientation of the sensor modules with respect to the body segments. The data was wirelessly transmitted to a laptop and recorded in MVN Studio version 3.4 at a sampling rate of 120 Hz. Accelerometer and gyroscope signals along with orientation and position data derived in MVN Studio [24] were exported to MATLAB R2013b (Mathworks, Inc., Natick, MA, USA) for further analysis. Typical acceleration and velocity waveforms of a subject walking and freezing are shown in Fig. 2. Step detection and gait cycle analysis were performed based on the SHOE zero velocity detection algorithm [27] and a non-linear walking algorithm [10].

### Statistical analysis

Statistical analyses were performed in R [29] using an alpha of 0.05 for all two-sided tests. The number of FOG episodes (per all trials with the same test condition) and the median FOG duration (s) were compared across different conditions using a Friedman rank sum test. Post hoc comparisons were performed with the Wilcoxon signed rank test with Bonferonni corrections.

Four gait parameters, namely cadence (steps/min), speed (m/s), mean stride length (m), and mean stride length variability (m), were compared across different tasks using multilevel analysis with the lme4 library [2]. Each parameter was modeled as a linear mixed model with two crossed within-patient factors (cue and walking course, each with four levels) and their interaction as fixed effects. Random intercepts and slopes for course and cue were included as random effects for each subject. For cadence, the cueing frequency selected by the patient was also added as a between-subjects fixed effect. Visual inspection of the residual plots did not reveal any obvious deviations from homoscedasticity or normality. Type III *F*-tests of fixed effects were performed using Satterthwaite’s approximation [25]. Random effects were tested using log-likelihood ratio tests. Post hoc testing with Bonferroni correction was conducted for significant effects or interactions using the glht function from the multcomp library [9]. Increases or decreases in the gait parameters during the cued trials compared to non-cued trials (control) were reported in terms of their mean ± standard error and percentage change of the mean.

### User experience

A short structured semi-open interview was conducted after the measurements to collect information on participants’ background with respect to mobile technology and cueing, their user experience with Glass and the cueing app (on a five point Likert scale), and suggestions for future implementations of the app. The specific questions are listed in the Appendix. Two independent raters categorized patient responses from voice recordings of the interviews and resolved any disagreements through discussion.

## Results

Visual inspection and multilevel modeling revealed distinct differences across subjects, walking courses, and cues. Notably, there was a high variability in FOG and gait performance within and amongst the participants. The effect of cueing on FOG and the gait parameters was highly dependent on the type of walking course. No practice effect was observed with increasing trials and no adverse events were observed during the trials.

### Freezing of gait

**Fig. 3:**
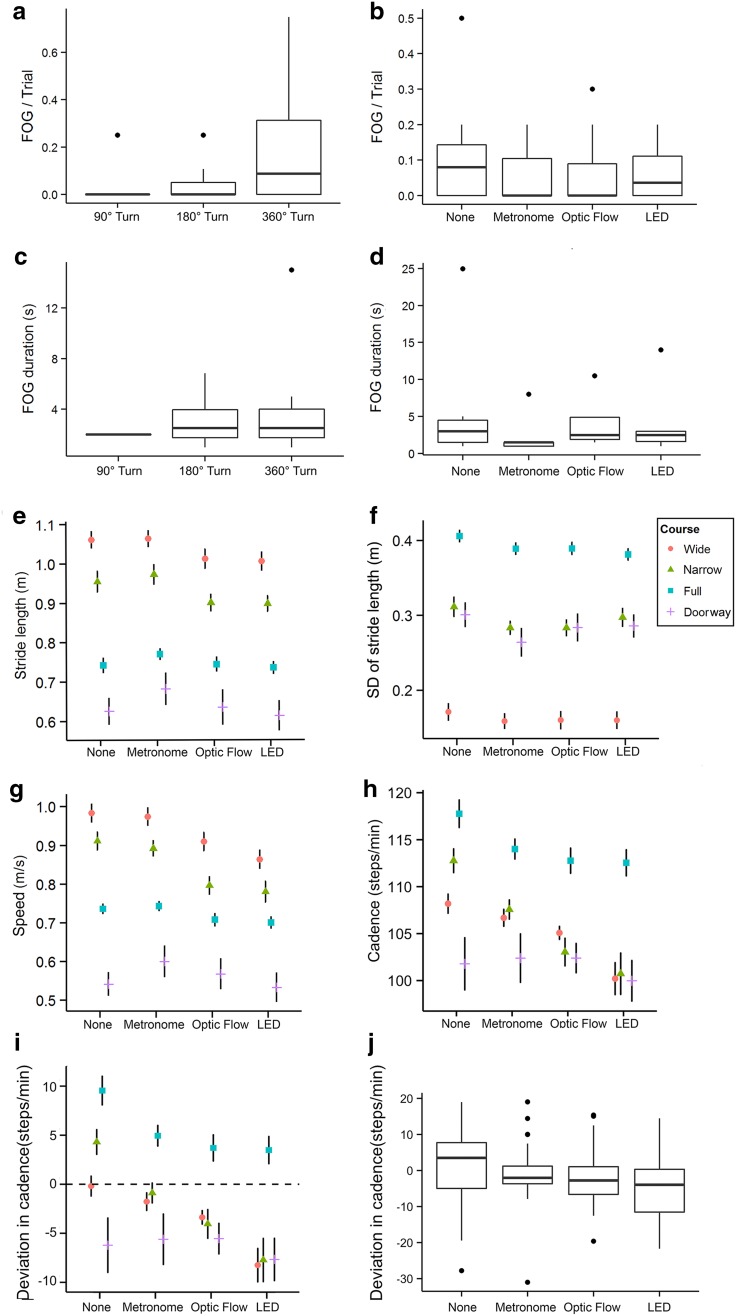
Effect of cueing on FOG and gait. **a**–**d** *Box-whisker plots* of the number of FOG episodes per trial (**a**, **b**) and their duration (**c**, **d**) for each type of turn (**a**, **c**) and cueing condition (**b**, **d**) (*N* = 12). **e**–**j** The stride length (**e**) and its standard deviation (SD) (**f**), speed (**g**), cadence (**h**), and deviation of the cadence from the cueing frequency (**i**, **j**) for different combinations of cues and walking courses in mean ± standard error (**e**–**i**) or as a *box-whisker plot* (**j**) (*N* = 11)

41 episodes of FOG were observed in eight out of 12 participants, six of whom experienced FOG more than once. The incidence of FOG differed across walking courses, with no FOG occurring on the wide turn course (Table 2). More specifically, for all cueing conditions, FOG was only observed during 90° turns, narrow 180° U-turns and 360° turns. No FOG occurred while subjects simply walked forward or performed wide 180° turns. FOG occurred in a higher percentage of participants and more frequently during 360° turns compared to narrow 180° turns (*z* = −2.29, *p* < 0.05, Fig. 3a), although the FOG duration did not significantly differ (*z* = 0.0, *p* > 0.05, Fig. 3c). The number of FOG episodes per trial (*χ*^2^(3) = 7.29, *p* = 0.063, Fig. 3b) and the FOG duration (*χ*^2^(3) = 2.42, *p* = 0.50, Fig. 3d) were not significantly different amongst cueing conditions. However, during 360° turns, fewer participants experienced FOG whilst using a cue and significantly less FOG episodes occurred per trial while using the metronome compared to no-cues (*p* < 0.05, *z* = −2.13).

**Table 2:** Number of patients (*N* = 12) who exhibited FOG for different combinations of cueing conditions and specific movements

	90° Turn	Narrow 180° turn	360° Turn
None	0	1	7
Metronome	0	2	5
Optic flow	0	2	3
LED	1	3	3

No FOG was detected during forward walking and wide 180° turns. 90° turns were only performed during the doorway course. Narrow 180° turns were present in the narrow turn, full turn, and doorway courses. 360° turns only occurred during the full turn course

### Stride length

There were significant main effects of the cue and course on both the stride length (cue: *F*(3,334) = 13.74, *p* < 0.001; course: *F*(3,11) = 86.57, *p* < 0.001, Fig. 3e) and stride length variability as measured by its standard deviation (cue: *F*(3,335) = 6.55, *p* < 0.001; course: *F*(3,11) = 180.47, *p* < 0.001, Fig. 3f). No significant interaction effects were found between the cue and course for either the stride length (*F*(9,326) = 1.28, *p* = 0.25) or its standard deviation (*F*(9,325) = 0.65, *p* = 0.75). Thus, the full models were simplified by eliminating the not significant interaction term and random slope for the cue. All cues showed a significant decrease in stride length variability in comparison to that for no-cues (metronome: −2.23 ± 0.56 cm (−7.1 %), *t*(335) = −3.97, *p* < 0.001; optic flow: −1.84 ± 0.56 cm (−5.9 %), *t*(335) = −3.30, *p* < 0.01; LED: −1.90 ± 0.56 cm (−6.1 %), *t*(335) = −3.38, *p* < 0.001). The metronome was associated with a significant increase in the stride length (2.22 ± 0.93 cm (2.6 %), *t*(334) = 2.38, *p* < 0.05) compared to no-cues while the optic flow (−2.44 ± 0.92 cm (−2.8 %), *t*(334) = −2.64, *p* < 0.01) and LED (−3.22 ± 0.93 cm (−3.8 %), *t*(334) = −3.45, *p* < 0.005) were associated with a decrease in stride length.

### Walking speed

We found significant effects of the cue (*F*(3,12) = 9.57, *p* < 0.01) and course (*F*(3,11) = 50.0, *p* < 0.001) on the walking speed (Fig. 3g) and significant interactions between these effects (*F*(9,314) = 5.08, *p* < 0.001). Compared to no-cues, the metronome was associated with a significant increase in speed only during the doorway course (5.62 ± 0.22 cm/s (11.2 %), *z* = 2.51, *p* < 0.05). In contrast, the speed significantly decreased during the wide and narrow turn courses for the optic flow (wide −7.33 ± 0.22 cm/s (−7.5 %), *z* = −3.34, *p* < 0.01; narrow: −9.72 ± 0.22 cm/s (−10.7 %), *z* = −4.42, *p* < 0.001) and LED (wide −11.92 ± 0.22 cm/s (−12.1 %), *z* = −5.321, *p* < 0.001; narrow: −13.1 ± 0.23 cm/s (−14.4 %), *z* = −5.79, *p* < 0.001). No significant differences were found for other cue-course combinations.

### Cadence

Significant main effects of the cue (*F*(3,12) = 4.38, *p* < 0.05), course (*F*(3,11) = 7.61, *p* < 0.01), and their interaction (*F*(9,322) = 3.49, *p* < 0.001) were observed on the cadence (Fig. 3h). The cueing frequency also had a significant effect (*F*(1,38) = 224.48, *p* < 0.001) on cadence. Replotting the main effects against the difference between the cadence and the cueing frequency, the effects of the cue and course can be interpreted as deviations of the cadence from the cueing frequency (Fig. 3i).

All cues showed significant decreases in cadence compared to no-cues for the narrow (metronome: −5.20 ± 1.75 steps/min (−4.6 %), *z* = −2.96, *p* < 0.01; optic flow: −8.25 ± 2.00 steps/min (−7.3 %), *z* = −4.12, *p* < 0.001; LED: −12.03 ± 2.35 steps/min (−10.7 %), *z* = −5.13, *p* < 0.001) and full turn courses (metronome: −4.35 ± 1.47 steps/min (−3.7 %), *z* = −2.97, *p* < 0.01; optic flow: −5.48 ± 1.78 steps/min (−4.7 %), *z* = −3.08, *p* < 0.01; LED: 5.17 ± 2.14 steps/min (−4.4 %), *z* = −2.41, *p* < 0.05). No significant effects were found for any cue during the doorway course. Only the LED was associated with a significant decrease in cadence during the wide turn course (−8.02 ± 2.33 steps/min (−7.4 %), *z* = −3.45, *p* < 0.01). Based on visual inspection of the data, the cadence tended to cluster around the cueing frequency for the three cues (Fig. 3i) and was less variable in its distribution for the metronome and optic flow (Fig. 3j).

### User experience

We report the number of total responses (*N*) per interview question, as not all participants provided an applicable response to every question. About half of the subjects reported a subjective improvement in walking while using cues (*N* = 5/10) and most were willing to use Glass at home against freezing of gait (*N* = 9/12). The metronome was most preferred (*N* = 11/12) while the optic flow was the least preferred (*N* = 11/12); only one patient preferred the LED to the metronome. The participants found it easiest to synchronize to the rhythm of the cues (*N* = 4) and to simultaneously walk (*N* = 1) whilst using the metronome. In contrast, patients reported it was difficult to synchronize to the optic flow (*N* = 4) and to walk while focusing on such cues (*N* = 1). They described the optical flow as annoying (*N* = 5), distracting (*N* = 4), demanding too much concentration (*N* = 3), and hard to see (*N* = 1). Nevertheless, participants reported that the cues were delivered at a comfortable speed (*N* = 7/9).

Most participants found Glass easy or very easy to use (*N* = 7/11) and the instructions on the screen clear or very clear to read (*N* = 9/12). Some had experience operating smartphones (*N* = 4) and tablets (*N* = 4), while others still used conventional mobile phones (*N* = 5). One participant particularly liked the bone-conducting headphone because the metronome was less audible to others around them. Conversely, some participants disliked Glass’ placement of the visual display in the upper right corner (*N* = 3) and suggested that images be projected binocularly (*N* = 1) or more focally (*N* = 2) in the visual field. Several subjects already used cues in their daily lives (*N* = 6), including the metronome (*N* = 3), laser pen or rollator that projects a laser stripe on the floor (*N* = 2), counting (*N* = 1), singing (*N* = 1), patterned floor tiles (*N* = 1), and verbal cues (*N* = 1). They suggested verbal instructions (*N* = 9), rhythmic music (*N* = 2), and postural feedback (*N* = 1) as additional cues for the app and that cues only be provided when needed (*N* = 2).

## Discussion

To investigate the potential of new mobile technologies as assistive devices, we developed a cueing application for Google Glass and evaluated its efficacy on improving gait performance in this preliminary feasibility study.

### Effects of cueing

Given that most participants exhibited mild FOG in the laboratory, we found no significant changes in the frequency and duration of FOG episodes under cueing conditions when all walking courses were considered together. However, in line with earlier studies [19, 23], a reduction in the number of FOG episodes was found using the metronome during complex 360° turns. Similar to previous reports [3, 28], sharper turns were the most potent strategies to induce FOG, although 360° turns are less likely to occur in daily life The difficulty of provoking FOG in laboratory settings is widely known [28]. Thus, studying cueing with portable devices at home is warranted.

We analyzed gait performance over the full trajectory in terms of the walking speed, cadence, and stride length (gait analysis on only turning was infeasible given the small number of steps within a turn). Cueing decreased cadence variability and significantly reduced stride length variability, which has been linked to the propensity for falling [26]. These findings suggest a more stable gait pattern with cueing, mediated by attentional strategies to normalize the stride length and cadence [1]. Further investigation is needed to ascertain whether cueing can achieve a lasting reduction of the stride length variability as a significant risk factor for future falls.

These gait parameters were not coupled in a fixed manner, with the complexity of the walking courses highly influencing the effectiveness of the cues. For instance, for the metronome, the stride length and walking speed increased the most during the more complex full turn and doorway courses. In contrast, visual cues were associated with a decrease in the stride length and speed during the simpler wide and narrow turn courses and no significant differences for the full turn and doorway courses. For both auditory and visual cues, the cadence decreased except during the doorway course, which is known to cause people with PD to slow down and provoke FOG [4]. These distinctions could be attributed to interference in gait performance during functional dual-tasks and external cueing reducing this interference during dual-motor tasks [23]. In this case, cueing may facilitate complicated tasks like walking while turning and entering a doorway whereas focusing on the cues may interfere with gait during simpler motor tasks like single task walking. Since visual cues were reportedly harder to focus on, potential improvements in gait may be diminished for complicated tasks and the decline worsened for simpler tasks.

We found that gait parameters improved more consistently with auditory cues than visual cues, in line with previous systematic reviews [12, 22]. Moreover, participants also preferred the metronome over the LED and optical flow, similar to the RESCUE project [19]. Placement of the display in the upper right corner of Glass may have diminished the potential benefits of visual cueing on focusing attention on gait and enhancing optic flow, as concentrating on the display while walking potentially created a visual dual-task. While rhythmic visual cues seemed to be less effective than auditory cues, spatial visual cues such as stripes on the floor have been shown to be effective in a laboratory setting [1, 31] but remain to be studied using smartglasses. Thus, it is unclear whether visual or auditory cues are more effective [12, 22].

### Limitations of the study

There are several limitations of the study. First, out of the 12 participants, only six experienced FOG more than once, four exhibited no FOG, and two had a single FOG episode. Due to this small sample size of freezers, the effect of cueing on FOG is inconclusive. Second, a potential confound is that many of the participants have already used cues in their daily life and may be more efficient during the cued walking trials. As the effects of cueing do not generalize well [19] and none of the participants had prior experience using the Google Glass, we do not expect that those with cueing experience would outperform those with no previous experience during this study. Visual inspection of individual performances also did not show consistent differences between these two groups. Third, as the study was conducted at end of dose, the findings may be less applicable to daily life when people are mostly in the on state. However, as FOG is known to be resistant to medication [18] and deep brain stimulation [11] and motor fluctuations—alterations between on and off states—are the most common complications of long-term levodopa use, cueing during the on state is still a useful strategy. Lastly, the version of the Google Glass used in this study is no longer available for purchase, with Google pursuing a new Enterprise edition of the Glass tailored for working environments. As numerous other augmented reality smart glasses are appearing on the market, mobile cueing will continue to advance.

### Future outlook

While the participants were overall positive about its user-friendliness, it is unclear whether Glass would be more effective in improving gait quality than cheaper conventional cueing modalities like the metronome. Clearly, further developments are necessary before Glass can be adopted for daily use. First, visual information should be projected binocularly or towards the center of the visual field to optimize the effects of visual cueing. Second, cues should ideally be personalized to activate the most appropriate alternative motor circuits [30] and cater to different needs and cueing preferences across people with PD [32]. Lastly, integration of automatic detection of FOG [16] and obstacles in the environment would facilitate cueing on a needs basis that would interfere less with their daily activities than continuous cueing. Thus, smartglasses have the potential to become mobile assistive devices for on-demand cueing in daily life, but further development is necessary to better accommodate the individual needs of people with PD [32].
